# The tricarboxylic acid cycle in *Shewanella oneidensis *is independent of Fur and RyhB control

**DOI:** 10.1186/1471-2180-10-264

**Published:** 2010-10-16

**Authors:** Yunfeng Yang, Lee Ann McCue, Andrea B Parsons, Sheng Feng, Jizhong Zhou

**Affiliations:** 1 Department of Environmental Science and Engineering, Tsinghua University, Beijing 100084, China; 2 Biosciences Division, Oak Ridge National Laboratory, Oak Ridge, TN 37831, USA; 3 Pacific Northwest National Laboratory, Richland, WA 99352, USA; 4 Department of Biostatistics and Bioinformatics, Duke University, Durham, NC 27710, USA; 5 Institute for Environmental Genomics and Department of Botany and Microbiology, University of Oklahoma, Norman, OK 73019, USA

## Abstract

**Background:**

It is well established in *E. coli *and *Vibrio cholerae *that strains harboring mutations in the ferric uptake regulator gene (*fur*) are unable to utilize tricarboxylic acid (TCA) compounds, due to the down-regulation of key TCA cycle enzymes, such as AcnA and SdhABCD. This down-regulation is mediated by a Fur-regulated small regulatory RNA named RyhB. It is unclear in the γ-proteobacterium *S. oneidensis *whether TCA is also regulated by Fur and RyhB.

**Results:**

In the present study, we showed that a *fur *deletion mutant of *S. oneidensis *could utilize TCA compounds. Consistently, expression of the TCA cycle genes *acnA *and *sdhA *was not down-regulated in the mutant. To explore this observation further, we identified a *ryhB *gene in *Shewanella *species and experimentally demonstrated the gene expression. Further experiments suggested that RyhB was up-regulated in *fur *mutant, but that AcnA and SdhA were not controlled by RyhB.

**Conclusions:**

These cumulative results delineate an important difference of the Fur-RyhB regulatory cycle between *S. oneidensis *and other γ-proteobacteria. This work represents a step forward for understanding the unique regulation in *S. oneidensis*.

## Background

Fur (Ferric uptake regulator) is a global transcription factor that regulates a diversity of biological processes such as iron homeostasis, TCA cycle metabolism, acid resistance, oxidative stress response, chemotaxis and pathogenesis (reviewed in [1]). The active, DNA-binding form of this regulator is as a Fur homodimer complexed with ferrous iron. The DNA target recognized by Fe^2+^-Fur is a 19-bp inverted repeat sequence called a "Fur box" (GATAATGATAATCATTATC) [2]. The binding of Fe^2+^-Fur to a "Fur box" in the promoter regions of target genes effectively prevents the recruitment of the RNA polymerase holoenzyme, and thus represses transcription [3,4].

Although Fur typically acts as a transcriptional repressor, it also appears to positively regulate certain genes in *E. coli *[5,6]. This paradox was understood only recently, with the discovery of a 90-nt small RNA named RyhB [7]. RyhB negatively regulates a number of target genes by base pairing with their mRNAs and recruiting RNaseE, thus causing degradation of the mRNAs [7,8]. The *ryhB *gene itself is repressed by Fur via a "Fur box" in its promoter; thus, Fur repression of the negative regulator RyhB manifests as indirect positive regulation by Fur. The targets of RyhB include genes encoding iron-storage protein (Bfr) and enzymes of the TCA cycle (SdhABCD and AcnA) and oxidative stress response (SodB) [7]. The RyhB-mediated regulation of TCA cycle genes explains the inability of *E. coli fur *mutants to grow on succinate or fumarate [9].

*S. oneidensis *is a γ-proteobacterium with a striking capacity to reduce organic compounds and heavy metals, making it a potential bioremediator of environmental contaminants. The *S. oneidensis *Fur exhibits clear homology to its *E. coli *ortholog (73% amino acid identity). Physiological, transcriptomics and proteomics studies have shown that *S. oneidensis *Fur regulates genes involved in iron homeostasis and acid resistance [10-13]. Consistently, many of these target genes have a recognizable "Fur box" in their promoters. In the present study, we further characterize a *fur *null mutant of *S. oneidensis *with regard to its ability to utilize succinate and fumarate. Unexpectedly, HPLC analysis showed that the *fur *mutant was able to metabolize succinate and fumarate, and the growth of the mutant was enhanced in the presence of succinate and fumarate, indicating that the mutant can utilize these compounds. In addition, the expression of the TCA cycle genes *acnA *and *sdhA *was not down-regulated in the mutant. These differences between *S. oneidensis *and *E. coli *were traced to the small RNA gene *ryhB*, which we identified in several *Shewanella *species. Although *S. oneidensis *RyhB was up-regulated in the *fur *mutant, the TCA cycle genes did not appear to be regulated by RyhB. These results delineate differences in the gene regulation and physiological consequences of RyhB between *S. oneidensis *and *E. coli*.

## Results

### TCA cycle activity and regulation in the *fur *mutant

We showed recently that *S. oneidensis *harboring a *fur *deletion in the genome was sensitive to acidic conditions and de-repressed genes encoding iron acquisition systems [11]. Similar observations have been made in *E. coli *[14,15], suggesting that the functional roles of Fur are conserved in these species. Since Fur acts as a pleiotropic transcription factor involved in multiple biological processes, we proceeded to examine its role in regulating TCA cycle enzymes. The involvement of Fur in this biological process has been established in *E. coli *and *V. cholerae *by observations that *fur *mutants are unable to grow in defined media with succinate or fumarate as a carbon source [9,16], and that genes encoding certain TCA cycle enzymes, such as succinate dehydrogenase (SdhABCD) and aconitase (AcnA), are significantly down-regulated in a *fur *mutant [7].

**Figure 1:**
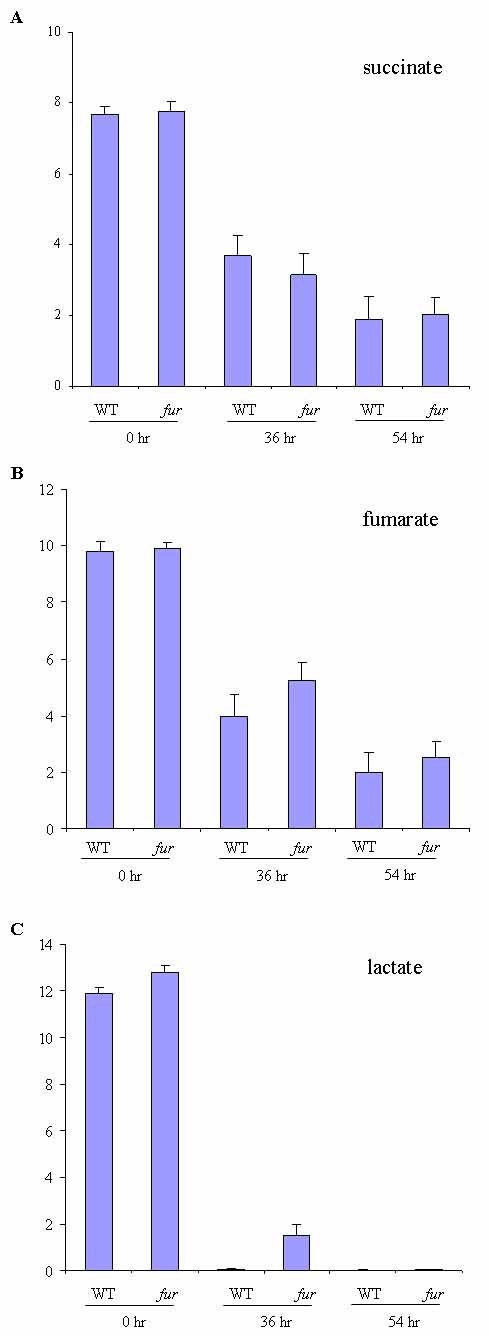
**Comparison of MR-1 and the *fur *mutant for their ability to metabolize carbonate: (A) succinate, (B) fumarate and (C) lactate**. 5 × 10^9 ^cells were incubated with 10 mM carbonate for 0, 36 and 54 hours. HPLC was used for carbonate measurements. Y-axis: the concentration of carbon source.

**Figure 2:**
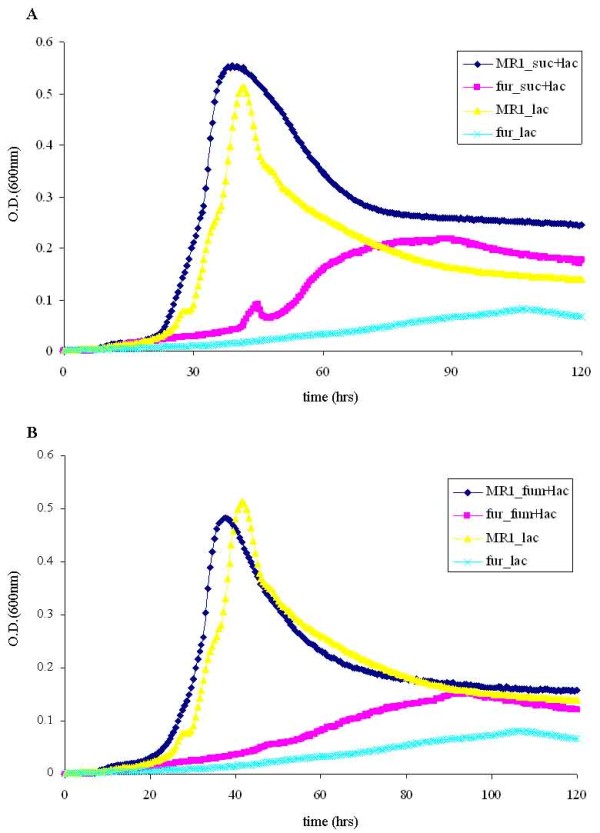
**The growth of wild-type (MR-1) and *fur *mutant in the presence of 10 mM lactate (lac) and (A) succinate (suc) or (B) fumarate (fum), which were supplied as carbon sources in defined medium**. Cell density was measured at OD_600 _every thirty minutes for five days. Data were averaged over triplicate samples.

Our initial tests showed that neither succinate nor fumarate, when provided as the sole carbon source in M1 defined media, could support detectable growth of *S. oneidensis *type strain MR-1 (data not shown), making it unlikely to analyze the growth of MR-1 and *fur *null mutant. However, the complete set of TCA genes is present in *S. oneidensis *genome, and recent studies have shown that the bacterium is capable of metabolizing succinate and fumarate [17,18]. To compare the metabolizing rates of the carbonates between MR-1 and the *fur *mutant, both strains were grown to mid-log phase with 10 mM lactate as the carbon source. Then equal numbers of cells (5 × 10^9^) were washed and resuspended in fresh M1 medium with 10 mM lactate, succinate or fumarate as the sole carbon source. Unexpectedly, HPLC analyses showed that both succinate and fumarate were reduced to similar levels in MR-1 and the *fur *mutant after 36 and 54 hours' incubation at 30°C (Figure 1A and Figure 1B), suggesting that *fur *mutant is not deficient in metabolizing succinate and fumarate. In contrast, more lactate was consumed in MR-1 than in the *fur *mutant (Figure 1C). This could be explained by the observation that there were more MR-1 cells after 36 hours' incubation (data not shown), as the MR-1 grew faster than the *fur *mutant when lactate was provided as carbon source (Figure 2). To determine whether the ability of the *fur *mutant in metabolizing succinate and fumarate affects cell growth, we grew MR-1 and the *fur *mutant in M1 medium with 10 mM lactate plus succinate or fumarate. Addition of succinate or fumarate significantly enhanced the growth of the *fur *mutant (Figure 2). Together, succinate and fumarate can indeed be similarly metabolized by MR-1 and the *fur *mutant of *S. oneidensis *and be used to support the cell growth when combined with lactate, though they are unable to support the cell growth as the sole carbon source.

A recent microarray study comparing the gene expression profile of the *fur *mutant to that of MR-1 showed that neither the *sdhCDAB *operon nor the *acnA *gene was down-regulated [11], which was unlike the observations in *E. coli*. To confirm this, quantitative RT-PCR was carried out on *acnA *and *sdhA*, a gene of the SdhCDAB operon. The housekeeping gene RecA was used as the internal standard to normalize the gene expression levels. The levels of SdhA and AcnA relative to RecA in MR-1 are 0.14 and 0.06, respectively. Both genes exhibited little change in expression in the *fur *mutant relative to MR-1 (Table 1). Therefore, the utilization of succinate or fumarate by the *fur *mutant (Figure 1) may be attributable to the persistent expression of TCA cycle genes. Notably, An putative iron uptake gene SO3032, which was expressed at the level of 0.04 relative to RecA in MR-1, was up-regulated in the *S. oneidensis fur *mutant. In contrast, the Fe-dependent superoxide dismutase encoded by *sodB*, a gene known to be regulated by Fur in *E. coli *[7], was repressed in the *fur *mutant (Table 1). This result agrees with previous observations that the transcript and protein expression levels of SodB are repressed in the *fur *mutant of *S. oneidensis *[10].

**Table 1:** Quantitative RT-PCR results.

Gene	*fur *mutant compared to MR-1	pFur/fur mutant compared to MR-1	pRyhB/*fur *mutant compared to vector/*fur *mutant	pRyhB/MR-1 compared to vector/MR-1
RyhB	20.1 (0.0006)	0.46 (0.07)	65.2 (0.0002)	61.4 (0.0001)
SdhA	1.06 (0.3)	0.89 (0.81)	1.07 (0.42)	1.56 (0.25)
AcnA	1.1 (0.42)	1.29 (0.63)	0.78 (0.44)	1.05 (0.47)
SodB	0.12 (0.03)	0.89 (0.57)	0.06 (0.01)	0.06 (0.008)
SO3032	16.7 (0.04)	2.32 (0.06)	N/A	N/A

The numbers in the cells are ratios of gene expression changes and the numbers in the parenthesis are *p *values of two-sided *t*-test. 0.05 is used as threshold to determine the significance of the changes.

### Identification of the small RNA RyhB in *Shewanella *species

**Figure 3:**
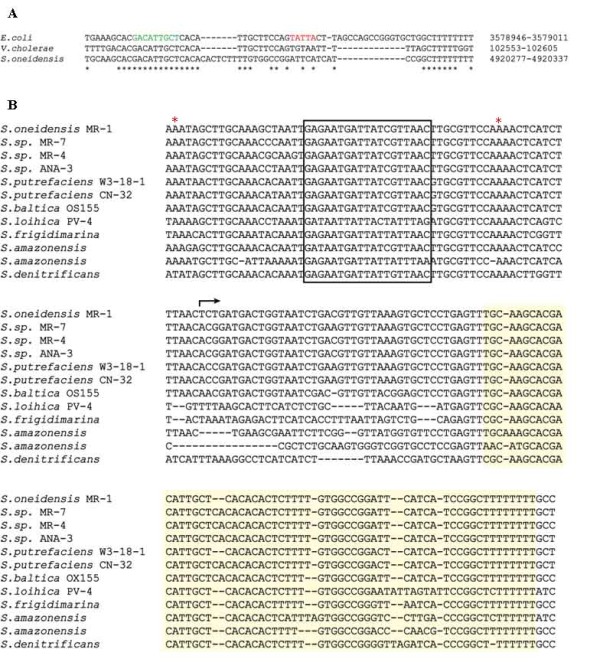
**Bioinformatics analyses of RyhB in *S. oneidensis*.** (A) Muscle multiple sequence alignment [39]**showing homology of the identified region of the *S. oneidensis *genome with the "core" region of *ryhB *from *E. coli *and *V. cholerae***. Genome coordinates for the sequences are from NC_000913 (*E. coli*), NC_002505 (*V. cholerae*), and NC004347 (*S. oneidensis*). The sequence shown in green is predicted to base pair with the *E. coli *SodB mRNA. The Hfq binding site is shown in red. (B) Muscle multiple sequence alignment of putative *ryhB *sequences from eleven species of *Shewanella*. The box indicates the conserved Fur binding site, the red stars are the start and end positions of the putative promoter, the bent arrow indicates the transcription start site for *S. oneidensis*, and the region highlighted in yellow is the region of RyhB shown in (A).

In *E. coli*, TCA cycle genes are controlled by a Fur-regulated small RNA named RyhB [7,19]. However, its homolog in *S. oneidensis *was not identified by homology to the *E. coli *RyhB using BLAST [20] or by searches using the *ryhB *sequence alignment and covariance model from Rfam [21]. Therefore, we examined the *S. oneidensis *MR-1 genome sequence in the region syntenic with the *V. cholerae *genomic region encoding RyhB. Specifically, the *V. cholerae ryhB *gene is located downstream of the gene VC0106 [22,23], which is orthologous (by reciprocal best-hit criteria) to the *S. oneidensis *gene SO4716. We identified a region downstream of SO4716 that exhibited homology with a region that was well-conserved among enterobacterial *ryhB *sequences (Figure 3A). This "core" region encompasses the sequence believed to base-pair with *E. coli sodB *mRNA and the binding site for the RNA chaperone Hfq [24].

**Figure 4:**
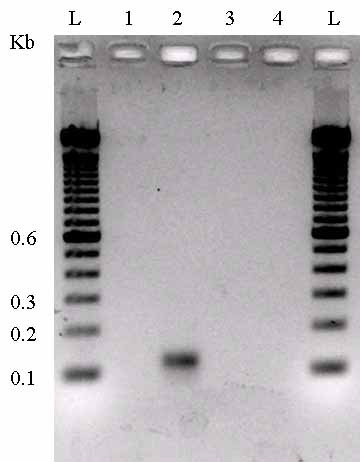
**Verification of the expression of small RNA RyhB by RT-PCR. L: DNA ladder; 1**. PCR amplification of *S. oneidensis *RNA without reverse transcription; 2. PCR amplification of sample after reverse transcription of RNA. The presence of the ~119 bp PCR product validates the expression of RyhB RNA. 3 and 4: PCR on two control intergenic regions (Chr. 367734-367820 and 796545-796665). The absence of PCR products indicates that genomic DNA has been completely removed from the RNA templates used for RT-PCR.

RT-PCR was performed to detect the expression of the putative RyhB transcript from this region of the *S. oneidensis *genome. Total RNA was prepared from wild type *S. oneidensis *MR-1 strain grown to mid-logarithmic phase and then used for reverse transcription-PCR. A PCR product with expected size of 119 bp was generated using *ryhB*-specific primers (Figure 4). This PCR product was absent when a PCR reaction was performed on RNA samples without reverse transcription, indicating that the RNA sample was free of genomic DNA contamination. This conclusion was also supported by the absence of PCR products when RT-PCR experiments were used to amplify six other intergenic regions (two examples are shown in Figure 4).

To determine the transcriptional boundaries of the RyhB transcript, 5'- and 3'-RACE experiments were carried out on the same sample used for RT-PCR, identifying a 168-nt transcript between nucleotides 4920234-4920401 of the *S. oneidensis *genome [25]. This transcript is longer than the 90-nt *E. coli *RyhB [19], but shorter than the 215-nt *V. cholerae *RyhB [22,23]. A "Fur box", matching 15 of the 19-base consensus sequence (GATAATGATAATCATTATC) [26], was predicted at positions -26 to -44 upstream of this gene (Figure 3B). Together, these results support the existence of a *ryhB *gene in *S. oneidensis*.

*ryhB *genes were subsequently identified in eleven other sequenced *Shewanella *species by BLASTN using the *S. oneidensis ryhB *sequence as the query. Extensive sequence conservation was observed (Figure 3B), including the "core" region identified as homologous with the enterobacterial *ryhB*. Two copies of *ryhB *were detected in the draft genome sequence of *S. amazonensis*, in a tandem arrangement similar to that observed for the *P. aeruginosa ryhB *[27]. The putative "Fur box" was also detected upstream of all of the *ryhB *homologs, suggesting that regulation of RyhB by Fur is a common feature among the *Shewanella *species.

### Over-expression of RyhB has no impact on the expression of TCA cycle genes

In *E. coli*, RyhB is highly up-regulated in a *fur *mutant, which in turn inhibits the expression of AcnA and SdhABCD enzymes and thus the TCA cycle. Since the expression of AcnA and SdhA remained unchanged in the *S. oneidensis fur *mutant, two possibilities exist as either RyhB is not regulated by Fur or that *acnA *and *sdhA *expression is independent of RyhB. To test the possibility that RyhB is not regulated by Fur, quantitative RT-PCR was performed to examine RyhB expression. As shown in Table 1, RyhB was induced 20-fold in the *fur *mutant. When the *fur *deletion was complemented by exogenous expression of Fur on the expression vector pBBR1MCS5-1, the RhyB induction was abolished (Table 1). In addition, regulation of RyhB by Fur was also supported by the identification of a "Fur box" upstream of RyhB (Figure 3B). To test the possibility that the expression of *acnA *and *sdhA *is independent of RyhB, *S. oneidensis *was transformed with a RyhB expression plasmid and quantitative RT-PCR performed. RyhB was 60-fold over-expressed relative to endogenous levels in MR-1 and the *fur *mutant (Table 1). Notably, the expression of SdhA and AcnA mRNAs remained unchanged by RyhB over-expression. In contrast, expression of the superoxide dismutase encoded by *sodB *was repressed, suggesting that the *S. oneidensis sodB *was negatively regulated by RyhB. In addition, over-expression of RyhB did not change the growth pattern of MR-1 or the *fur *mutant in the presence of succinate or fumarate (data not shown). Together, these results suggest that negative regulation of RyhB by Fur exists in *S. oneidensis*, but *sdhA *and *acnA *are not part of Fur-RyhB regulon. Therefore, the TCA cycle in *S. oneidensis *is independent of Fur and RyhB control.

## Discussion

It is of interest to note that succinate and fumarate cannot support the growth of MR-1. Genomics analysis indicates that MR-1 contain the complete gene set required for TCA cycle. However, a recent metabolic flux analysis [17] showed that the anaplerotic pathway (Pyr → Mal) and (Pyr → PEP) were unidirectional, indicating that succinate and fumarate could not be used to produce pyruvate and Acetyl-CoA. Since Acetyl-CoA is the precursor of critical biomass components such as lipids, the inability to convert succinate and fumarate into Acetyl-CoA leads to the growth inhibition of MR-1. In contrast, lactate could be metabolized into pyruvate as well as other central metabolites and thus supports the cell growth.

The inability of *E. coli fur *mutant to grow on succinate or fumarate has been attributed to the down-regulation of *acnA *and *sdhCDAB *by the Fur-regulated small RNA, RyhB [7]. However, this regulatory mechanism of TCA cycle is not present in the γ-proteobacterium *S. oneidensis*, as evidenced by three observations: (1) both microarray and quantitative RT-PCR experiments showed that expression of *acnA *and *sdhA *remained unchanged in the *fur *mutant; (2) MR-1 and the *fur *mutant showed similar reduction of succinate and fumarate; and (3) succinate or fumarate enhanced the growth of the *fur *mutant. To explain the observations, we showed that although *S. oneidensis *RyhB was up-regulated in the *fur *mutant, over-expressing RyhB caused little change in the expression of *acnA *and *sdhA *as well as the growth with succinate or fumarate. Therefore, *acnA *and *sdhA *are not part of the Fur-RyhB regulon in *S. oneidensis*.

Intriguingly, we found that over-expressing RyhB enhanced the growth of the *fur *mutant in LB medium containing iron chelator (unpublished data), suggesting that RyhB plays a role in iron response of *S. oneidensis*. However, additional work is needed to delineate the regulon of RyhB and its regulatory mechanism.

**Figure 5:**
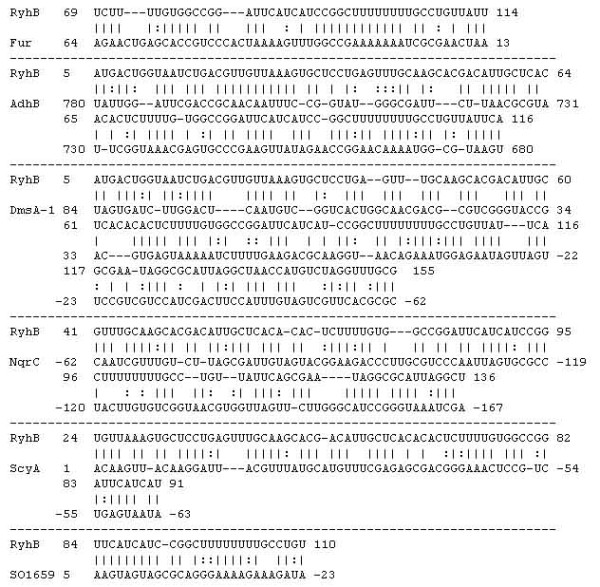
**Complementarity between RyhB and its potential targets**. The alignment shows the predicted interaction between RyhB and the anti-sense strand of target genes. The numbers represent the start and end positions of the nucleotides. All of the base pairing is considered significant, as judged by *p *value less than 0.01 [29].

RyhB acts as a post-transcriptional regulator by base pairing with its target mRNAs [7]. Therefore, it is possible to predict its direct targets by surveying DNA sequences for possible base-pairing. A likely target is the SodB mRNA, as evidenced by the presence of sequences in the "core" region of *Shewanella *RyhB that could potentially base-pair with SodB mRNA [24] and the repression of *sodB *in strains over-expressing RyhB (Table 1). No likely base pairing between RyhB and *sdhCDAB *or *acnA *was noted by manual inspection or by computational programs designed to predict small RNA targets [28,29]. Worthy of mention is that a program called TargetRNA [29] identified possible base pairing between *ryhB *and Fur genes (Figure 5), implying the possibility of a regulatory feedback loop. Such a regulatory circuit has recently been verified in *E. coli *[30]. In addition, several genes involved in anaerobic respiration, such as those encoding alcohol dehydrogenase II (AdhB), anaerobic DMSO reductase (DmsA-1), NADH:ubiquinone oxidoreductase subunit (NqrC-2) and two *c*-type cytochromes (ScyA & SO1659), possess extensive complementary regions with *ryhB *(Figure 5). Although interesting, these predictions require experimental validation involving a *ryhB *null mutant. Nevertheless, we have not been able to generate the mutant despite of multiple attempts, which might be attributed to technical difficulties or the possibility that *ryhB *is an essential gene in *S. oneidensis*.

The differences we observed in the RyhB regulon, relative to that of *E. coli*, are perhaps not surprising in light of the low level of sequence conservation among *ryhB *genes in phylogenetically related bacteria, implying that *ryhB *evolves at a rapid pace. Thus far, the only persistent structural features among the known *ryhB *homologs are the presence of an upstream Fur binding site and a region complementary to the SodB mRNA. The former has been employed to identify *ryhB *in *P. aeruginosa *[27].

Accumulating evidence suggests that regulatory pathways in *S. oneidensis *are distinct from other γ-proteobacteria. For example, the *E. coli *cAMP receptor protein (CRP) controls the transcription of a number of catabolic genes, but its *S. oneidensis *homolog is involved in regulation of anaerobic respiration [31]. Also, a major regulator of anaerobic respiration in *E. coil *(FNR) shows little involvement in anaerobic respiration in *S. oneidensis *[32-34]. Furthermore, the regulons of the global regulators ArcA and Fur are clearly distinct from that in other bacteria despite significant overlap [10,35].

## Conclusions

In accordance with current findings of distinct gene regulatory pathways in *S. oneidensis*, our study provides evidence to delineate the unique RhyB gene regulation in *S. oneidensis*.

## Methods

### Growth conditions and strain construction

M1 defined medium [36] was used. Cell growth was measured by a type FP-1100-C Bioscreen C machine (Thermo Labsystems) at 600 nm after growing cells to mid-logarithmic phase and diluting 1:100 into 300 μl fresh medium. Triplicate cultures were used to determine average and standard deviation. To prepare cultures for real time PCR (RT-PCR), strains were inoculated into 20-50 ml medium in 250 ml flasks. The cultures were incubated at 30°C with vigorous shaking (250 rpm). When the cultures reached mid-logarithmic phase, the cells were collected by centrifugation and flash frozen in liquid nitrogen. Cells were stored at -80°C prior to RNA extraction.

For exogenous expression of Fur and RyhB, the *fur *and *ryhB *open reading frames (ORFs) were PCR amplified with primers fur-F1 and fur-R1, and ryhB-F1 and ryhB-R1, respectively (Table 2). The PCR products were digested with SalI and EcoRI, and cloned into the broad-range expression vector pBBR1MCS5-1 (Km^r^), placing the ORFs under the transcriptional control of a strong *lac *promoter. The resulting plasmids were verified by DNA sequencing and transferred into *E. coli *WM3064, which is a diaminopimelic acid (DAP) auxotroph with plasmid RK4 integrated in the chromosome to mobilize plasmid *in trans *during conjugation [37]. Conjugation was carried out by mating *E. coli *and *S. oneidensis *in 1:1 donor/recipient ratio for 8 hrs on a LB/DAP plate at 30°C followed by selection of *S. oneidensis *transconjugants on LB agar plates supplemented with 50 μg/ml kanamycin. The vector pBBR1MCS5-1 was also transformed into *S. oneidensis *for the purpose of comparison.

**Table 2:** Oligonucleotide primers used in this study.

Primer name	Sequence
**strain construction**	
fur-F1	GGTCGACCAAGAGATTAGCAATGACAGATG
fur-R1	GGAATTCGAGCAAGCTTATTCGTCGT
ryhB-F1	GGTCGACAGGAGGAACTCTGATGACTGGTAATCTG
ryhB-R1	GGAATTCAGTTAAATGTGGCGCAAAC
**Reverse Transcription-PCR**	
ryhB-F2	TCTGACGTTGTTAAAGTGCTCC
ryhB-R2	CCTAATGCGCCTATTCGCT
Control 1-F	TCAGGTTGTTTGGTATTGTGC
Control 1-R	CCATCAATCAAGGTTGTCG
Control 2-F	CTGTCAAATGGTGTGCTGC
Control 2-R	GTGTAACAGTGCTAAAGCCTGC
Control 3-F	TCTACTCAAATGACGAGCTGC
Control 3-R	GAAAAGCCGCCAAATGC
Control 4-F	TATGGTTTCCCGCTTTCG
Control 4-R	AACGCATCAGTGCTATTTGC
Control 5-F	TCACTCACAGAACGCTTCG
Control 5-R	GCAGCTACAGAATGTCACTACG
Control 6-F	TCTAGCAGGGATTAAATGAGC
Control 6-R	CCTTCGCCTTGTCTAAAGC
**5'- and 3'-RACE assays**	
5'- RNA adapter	GAUAUGCGCGAAUUCCUGUAGAACGAACACUAGAAGAAA
ryhB-R3	AGAGTGTGTGAGCAATGTCG
3'- RNA adapter	UUCACU GUUCUUAGCGGCCGCAUGCUC-idT
**Quantitative RT-PCR**	
RyhB-F	TCTGACGTTGTTAAAGTGCTCC
RyhB-R	CCTAATGCGCCTATTCGCT
SdhA-F	GAGCAGTTAAAAGCCATCC
SdhA-R	GTTGTCCAATTCTAAACACTCG
AcnA-F	ACCAACAAACGCTAGACTACC
AcnA-R	ATCATCGCTCCACAAACC
SodB-F	TCTACTGGAACTGCTTAGCACC
SodB-R	TGAATGCATCGAATGAACC
RecA-F	AACCCAGAAACCACAACG
RecA-R	ACCAACCACCTCATCACC

Primer sequences were derived from the *S. oneidensis *MR-1 genome sequence [25]. F and R stand for forward and reverse primers, respectively.

### HPLC analyses

*S. oneidensis *wild-type (strain MR-1) and the *fur *mutant were grown to mid-logarithmic phase in M1 medium with 10 mM lactate as the sole carbon source. Cell density was determined by cell counting under microscope and 5 × 10^9 ^cells were collected by centrifugation, washed three times with PBS, and inoculated into 3 ml of fresh M1 medium with 10 mM of one of the following carbon sources: lactate, succinate or fumarate. These cultures were incubated at 30°C with vigorous shaking, and at time 0, 36 and 54 hrs, 1 ml culture was centrifuged. The supernatant was used for HPLC with an Elite LaChrom system (Hitachi). The samples were filtered with PALL Life Science Acrodisc 13 mm syringe filters with 0.2 μm nylon membranes, and analyzed with 5 mM H_2_S0_4 _mobile phase filtered with Gelman Sciences Nylaflo 47 mm 0.45 μm nylon membrane filter paper, degassed and at 0.5 mL/min flowrate for 35 mins with Biorad -Aminex HPX-87H column (300 × 7.8). The column temperature was maintained at 60°C, and the RI detector maintained at 50°C.

### RNA isolation and Reverse Transcription-PCR

Total cellular RNA was isolated using the TRIzol reagent (Invitrogen) according to the manufacturer's instructions. RNA samples were treated with RNase-free DNase I (Ambion) to digest residual chromosomal DNA and purified with RNeasy Kit (Qiagen) prior to spectrophotometric quantification at 260 nm. For RT-PCR, 0.1 μg RNA template was used in a Superscript One-step RT-PCR kit (Invitrogen) as recommended by the manufacturer. The primers used were ryhB-F2 and R2, control 1-6 F and R (Table 2).

### 5'- and 3'-RACE assays

RACE (rapid amplification of cDNA ends) experiments were carried out essentially as described [19]. For 5' RACE, the 5'-triphosphates of 15 μg total RNAs were converted to monophosphates by 25 units of tobacco acid pyrophosphatase (Epicentre Technologies) at 37°C for 1 hr, followed by phenol/chloroform extraction and ethanol precipitation. Precipitated RNA was resuspended in water and ligated to 500 pmol 5'- RNA adapter (Table 2). The ligated product was purified by phenol/chloroform extraction and ethanol precipitation, and reverse transcribed with 2 pmol sRNA-specific primer RyhB-R3 using the Thermoscript RT system (Invitrogen). The product was amplified by PCR, cloned into a pCR2.1 TOPO vector (Invitrogen) and sequenced. 3'-RACE assays were performed similarly to 5'-RACE, except that total RNA was dephosphorylated by calf intestine alkaline phosphatase (New England Biolabs), ligated to a 3'-RNA adapter (Table 2) and reverse transcribed with 100 pmol of a single primer complementary to the 3'-RNA adapter.

### Quantitative RT-PCR

The cDNA template for RT-PCR was synthesized in a 10 μl final reaction volume containing 3 μg of total RNA, 3 μg random primers (Invitrogen), 0.5 μM dNTPs, 10 mM DTT, 1 × first-strand buffer and 100 U of Superscript II reverse transcriptase (Invitrogen). After incubation at 42°C for 2 hours, the reaction was diluted five fold in H_2_O and stored at -80°C. Quantitative RT-PCR was carried out in an iCycler thermal cycler (Bio-Rad) in a 30 μl reaction mixture containing 15 μl iQ SYBR supermix (Molecular Probes), 1 μl cDNA template, and 160 nM forward and reverse primers. Primers were designed using the program Omiga 2.0 (Oxford Molecular) to yield a PCR product of ~100 bp in length (Table 2). Four technical replicates were performed for each of at least three biological replicates. The house-keeping gene *recA *was used as an internal control. That is, all results were normalized to the *recA *results obtained in parallel on the same sample to adjust for variation introduced during reverse transcription and RT-PCR. Specifically, the expression values were normalized by subtracting the mean of the *recA *expression values of the same samples. Different sources of variation (e.g. biological and technical replicates) were accounted for by linear mixed models [38]. The significance of the ratios between two samples was determined using a two-sided *t*-test, with a type 1 error of 0.05.

## Authors' contributions

YY conceived the study, implemented experiments to identify *ryhB *and drafted the manuscript. LAM performed bioinformatics analyses and manuscript editing. ABP carried out quantitative RT-PCR and growth experiments and performed manuscript editing. SF performed statistical analyses. JZ coordinated the study and performed manuscript editing. All authors have read and approved the manuscript.
